# MicroRNAs regulating macrophages infected with *Leishmania L.* (*V.*) *Braziliensis* isolated from different clinical forms of American tegumentary leishmaniasis

**DOI:** 10.3389/fimmu.2023.1280949

**Published:** 2023-12-07

**Authors:** Tainã Lago, Lilian Medina, Jamile Lago, Nadja Santana, Thiago Cardoso, Alan Rocha, Thyago Leal-Calvo, Edgar M. Carvalho, Léa Cristina Castellucci

**Affiliations:** ^1^ Serviço de Imunologia da Universidade Federal da Bahia, Salvador, Brazil; ^2^ Programa de Pós-graduação em Ciências da Saúde da Universidade Federal da Bahia, Salvador, Brazil; ^3^ Instituto Nacional de Ciência e Tecnologia em Doenças Tropicais (INCT-DT), Ministério da Ciência, Tecnologia, Inovações e Comunicações, CNPq, Brasília, DF, Brazil; ^4^ Laboratório de Pesquisas Clínicas (LAPEC), Instituto Gonçalo Moniz-FIOCRUZ, Sakvador, Bahia, Brazil; ^5^ Fundação Oswaldo Cruz, Fiocruz, Rio de Janeiro, BA, Brazil

**Keywords:** leishmaniasis, macrophages, genes, miRNAs, parasite load

## Abstract

**Background:**

Leishmaniasis is an infectious disease caused by protozoa of the genus *Leishmania*. There are still no vaccines, and therapeutic options are limited, indicating the constant need to understand the fine mechanisms of its pathophysiology. An approach that has been explored in leishmaniasis is the participation of microRNAs (miRNAs), a class of small non-coding RNAs that act, in most cases, to repress gene expression. miRNAs play a role in the complex and plastic interaction between the host and pathogens, either as part of the host’s immune response to neutralize infection or as a molecular strategy employed by the pathogen to modulate host pathways to its own benefit.

**Methods:**

Monocyte-derived macrophages from healthy subjects were infected with isolates of three clinical forms of *L. braziliensis*: cutaneous (CL), mucosal (ML), and disseminated (DL) leishmaniasis. We compared the expression of miRNAs that take part in the TLR/NFkB pathways. Correlations with parasite load as well as immune parameters were analyzed.

**Results:**

miRNAs -103a-3p, -21-3p, 125a-3p -155-5p, -146a-5p, -132- 5p, and -147a were differentially expressed in the metastatic ML and DL forms, and there was a direct correlation between miRNAs -103a-3p, -21-3p, -155-5p, -146a-5p, -132-5p, and -9-3p and parasite load with ML and DL isolates. We also found a correlation between the expression of miR-21-3p and miR-146a-5p with the antiapoptotic gene *BCL2* and the increase of viable cells, whereas miR-147a was indirectly correlated with CXCL-9 levels.

**Conclusion:**

The expression of miRNAs is strongly correlated with the parasite load and the inflammatory response, suggesting the participation of these molecules in the pathogenesis of the different clinical forms of *L. braziliensis*.

## 1 Introduction

According to the World Health Organization, there are three main forms of leishmaniasis: visceral (VL), the most serious form because it can be fatal if untreated; cutaneous (CL), the most common disease, usually causing skin ulcers; and mucocutaneous (ML), affecting mouth, nose, and throat (1). Given the complexity of CL presentation in South America, we will therefore use the term American tegumentary leishmaniasis (ATL) in order to describe these clinical forms. ATL extends from Mexico to Argentina (2) and is caused by various species of the *Leishmania (Viannia) braziliensis* and *Leishmania mexicana* complexes of parasites (3). *Leishmania* spp. mainly infect and survive in macrophages, but other cell types such as dendritic cells, lymphocytes, NK cells, and neutrophils participate in disease pathogenesis (4, 5). *Leishmania (Viannia) braziliensis* is the main species causing ATL in Brazil and can provoke cutaneous leishmaniasis (CL), characterized by one or more granulomatous ulcers, to the debilitating mucosal (ML) or disseminated (DL) forms of the disease (6–11). ML presents many distinct manifestations, from lesions limited to the nasal and oral cavity (mild stage), involvement of the epiglottis (moderate stage), to the involvement of the vocal cords, subglottic region, trachea, and even bronchi (severe stage) (12). DL is a severe and emerging form of ATL, defined by the presence of more than 10 polymorphic cutaneous lesions, distributed over more than two noncontiguous parts of the body. Nasal mucosal involvement is frequent, and the therapeutic failure rate is about 75%. DL development is related to a complex network involving environmental, host immune response, and parasite factors. It is important to distinguish DL from the anergic diffuse form—a very rare form of ATL caused by *L. amazonensis*, and from atypical manifestations, common in immunosuppressed patients who have multiple cutaneous lesions (13). Toll-like receptors (TLR) are used by several cell types in the recognition, internalization, and processing of antigens working as a link between the innate and adaptive immune response (14, 15). Data have demonstrated key interactions between macrophages and *Leishmania* through TLR2, TLR4, and TLR9 (16, 17). In CL, infection with *L. braziliensis* specifically increases the expression of TLR2 and TLR4 (18, 19), strengthening TLR signaling as an important layer of the host–pathogen interaction that will culminate in both inflammatory and healing responses. These fine mechanisms are regulated by the action of epigenetic factors, such as miRNAs, which represent a well-characterized class of non-coding RNA molecules of ~22 nucleotides. These molecules target messenger RNA (mRNA) by inhibiting its translation and, subsequently, protein synthesis (20, 21). In leishmaniasis, data showed alterations in the expression of miRNAs in macrophages infected by *L. major* after signaling of TLRs (22). Furthermore, there was a species-specific down-regulation in the expression of host cell miRNAs after infection with *L. major* and *L. donovani* (23). In another study, miR-193b, miR-671, and TREM1 were correlated with faster wound healing in patients infected with *L. braziliensis* (24). In addition, we documented that miR-361-3p was significantly more expressed in CL lesions caused by *L. braziliensis* than in normal skin and that this miR expression was also associated with failure of antimonial therapy and, consequently, prolonged healing time of cutaneous ulcers (25). In this work, we evaluated the expression of miRNAs acting on the TLR/NFKB pathway in macrophages infected with isolates of *L. braziliensis* derived from patients with different forms of ATL (CL, ML, and DL) from an endemic site of northeastern Brazil.

## 2 Material and methods

### 2.1 Culture of *L. braziliensis* isolates

Isolates of *L. braziliensis* (BR/18627, BR/30028, BR/30035) were obtained from skin and mucosal lesions of patients with CL, ML, and DL from the endemic area of Corte de Pedra, Bahia, Brazil. The parasites were initially cultured in biphasic medium NNN—Neal, Novy, and Nicolle (modified blood agar) and LIT (Liver Infusion Tryptose). Following isolation, the parasites were cryopreserved in frozen nitrogen. The isolates were expanded in Schneider medium supplemented with 10% fetal bovine serum (FCS) and 2% urine, and cultivated in a CO2 oven at 24°C. For seven days, the growth cycle of the parasite was monitored by counting viable promastigotes in order to assess the different stages of *L. braziliensis* growth until the stationary phase.

### 2.2 Obtaining macrophages from PBMC

Ten healthy volunteers of both sexes, aged between 25 and 50 years, were recruited from the staff of the Immunology Service at the Federal University of Bahia, where the study was conducted. As a non-endemic site, these people were not tested for present or previous history of the disease; however, all of them reported verbally that they had never had leishmaniasis or had lived in an endemic site. Peripheral blood mononuclear cells (PBMCs) were obtained from heparinized blood using Ficoll Hypaque™ Plus density gradient (GE Healthcare, Biosciences AB Durham, NC, USA), as previously described (26, 27). Briefly, cells were incubated in Teflon flasks for 6 days at 37°C and 5% CO2 to differentiate monocytes into macrophages. After this incubation period, the cells were washed and adjusted to 1x10^6^ and distributed in duplicate Lab-Tek slide wells and in 12-well culture plates. Subsequently, the cells were incubated for 24 hours for the macrophages to adhere to the plate prior to saline wash. Cultured cells were identified as macrophages by microscopic observation. Due to the practice we used in this protocol, we chose not to add growth factors to the culture, as these could influence the expression of miRNAs. However, this component can be added to improve the performance of obtaining monocyte-derived macrophages (MDMs).

### 2.3 Infection of macrophages with isolates of *L. braziliensis*

Macrophage cultures (1x10^6^ cells/well) were infected with *L. braziliensis* promastigotes in the stationary phase at a ratio of 5:1, in duplicate, for 48 hours, and uninfected macrophages were used as controls. The infection rate was evaluated by optical microscopy, counting the percentage of infected macrophages and the number of amastigotes per 200/cells in duplicate, for 4 hours only, which would be enough time to guarantee the entry of the parasites into the macrophages. We used the Panótico Rápido (Laborclin, Brasil) kit, a rapid staining set in hematology that contains dyes with chemical characteristics and affinities.

### 2.4 RNA extraction, cDNA synthesis, and evaluation of miRNA expression by quantitative RT-PCR

Based on previous work done with infected cells (22, 28), we evaluated the expression of miRNAs 4, 12, and 24 hours after parasite exposure, considering that their expression should be optimal in the first hours post-infection. The cell RNA was extracted using the TRIzol^®^ Reagent method, according to the manufacturer’s instructions. The isolated RNA was resuspended in 25μl of RNAse-free water, and the concentration was determined by optical density measurements (260 and 280 nm) using Nanodrop^®^. The miRNAs -146a-5p, -146b-5p, -147a, -155-5p, -9-3p, -125a-3p, -132-5p, 21-3p, hsa-let-7a-3p, -511-3p, -103a-3p, and the U6snComplementary DNA (cDNA) were chosen according to their contribution to the TLR activation pathway according to the literature and websites for TargetScanHuman (release 7.1; http://www.targetscan.org/) and miRBase (release 21; http://www.mirbase.org/). cDNA was obtained using the miRCURY LNA™ RT Kit (QIAGEN), following the manufacturer’s guidelines. The gene expression was performed using qPCR, using the miRCURY LNA SYBR Kit. Samples were prepared in duplicates as follows: 5µl of 2x miRCURY LNA SYBR Green Master mix, 0.05µl of ROX Reference Dye, 1µl of PCR primer mix, and 1µl of RNase-free water. A final amount of 10µl was used for each reaction, containing 3µl of the diluted cDNA template (1:5) and 7µl of the reaction mix. The amplification conditions followed the following cycling: (I) 2 minutes at 95°C, (II) 10 seconds at 95°C, (III) 60 seconds at 56°C, and (IV) for 40 cycles following the melting curve. For normalization, the threshold cycle (Ct) values of the expression of each miRNA were used. The expression of the evaluated miRNAs was normalized by miR-146b-5p miRNAs and by corresponding U6 (nucleolar RNA) for each sample in order to control for variability between samples. After normalization, gene expression units were calculated from values of ΔCt.

### 2.5 cDNA synthesis and gene expression of BCL2, CFLAR, ATG12, GZMB, and TNFRSF10A

In order to choose genes related to cell death processes and which were regulated by miRNAs previously associated with the ML and DL forms in the study, we consulted the notes deposited in the TargetScan database. TaqMan™ assays containing specific primers and probes for the *BCL2* (Hs01048932_g1), *CFLAR* (Hs01048932_g1), *ATG12*, (Hs00269492_m1), *GZMB* (Hs00188051_m1), and *TNFRSF10A* (Hs00269492_m1) genes were purchased from Thermo Fisher Scientific as well as the normalizer for the *ACTB* gene (Hs99999903_m1). Reverse transcription reactions were performed using the commercially available High-Capacity cDNA Reverse Transcription Kit (Thermo Fisher Scientific), starting from 10µl of total RNA and using the MultiScribe Reverse Transcriptase enzyme, according to the manufacturer’s instructions. Gene expression analysis was performed using the qPCR technique with the QuantStudio 3 (Applied Biosystems^®^). All samples were prepared in duplicate using the reagents recommended by the manufacturer. Data were analyzed using the Ct comparative method, according to the 2^-^ **^ΔΔ^** ^CT^ equation, where **Δ**CT is the Ct value of the target gene subtracted from the Ct of the endogenous gene, and the ΔΔCT is the ΔCT value of each individual, minus the median ΔCT of the control group.

### 2.6 Evaluation of cell death and oxidative burst

Flow cytometry was used to access apoptotic/necrotic cells and determine nitric oxide (NOS) and reactive oxygen species (ROS) expression. At 24 and 48 hours, cultured cells were labeled with 5 µL PE–anti-CD14 MAb (clone 61D3; BD Bioscience). Afterwards, 5 µL of annexin V (AnV) FITC in annexin binding buffer (BD Bioscience PharMingen) was added for 20 min. Samples were analyzed on a FACS Fortessa flow cytometer (BD Bioscience PharMingen, San Jose, CA). To access NOS and ROS intracellular species, cells were labeled with dihydrorhodamine 123, DAF-FM diacetate (Life Technologies), dihydroethidium (Life Technologies), and CM-H2DCFDA (Invitrogen—Thermo Fisher Scientific), diluted according to the manufacturer’s instructions. Viable cells (AnV-/IP-) after 48 hours were counted. A minimum of 50,000 gated events from each sample were collected in a FACS Fortessa flow cytometer (BD Bioscience PharMingen, San Jose, CA) and analyzed using the FlowJo 7.6.5 program.

### 2.7 Cytokine and chemokine production in macrophage culture supernatants

Cytokine and chemokine were measured in the supernatant of macrophages infected with *L. braziliensis* isolates at 24 hours after infection by the sandwich ELISA immunoenzymatic technique, using the commercially available R&D Systems Kit (R&D Systems). The levels of chemokines/cytokines CXCL-9, CXCL-10, TNF, IFN-γ, IL-6, IL-1β, and IL-10 were measured using the protocol recommended by the manufacturer.

### 2.8 Statistical analysis

We used Refinder (https://www.heartcure.com.au/reffinder/?type=reference) to analyze the stability of miRNAs expression. Refinder integrates computer programs available for normalization (geNorm, Normfinder, BestKeeper) and the comparative delta-Ct method to rank candidate reference genes tested among the different samples. In our study, U6snRNA and miR-146b-5p were identified as normalizing genes. After normalizing the genes, the miRNA expression data were represented in relative expression units between the CL, ML, and DL. Then, within each time point, conditions were tested against each other through the mixed linear model with random effect for individuals, followed by Tukey’s test for multiple comparisons. Correlation analysis with immune markers, parasite load, and gene expression was performed using the Spearman correlation test implemented by the GraphPad Prism 8 software. *p*-values < 0.05 were considered statistically significant.

### 2.9 Ethical statement

The study was approved by the institutional review board of the Federal University of Bahia (CAAE: 93214818.2.0000.0049) and according to the recommendations of Resolution 466/12 of the National Health Council for research with human beings and the Declaration of Helsinki.

## 3 Results

### 3.1 Infection of human macrophages with strains of *L. braziliensis*

**Figure 1:**
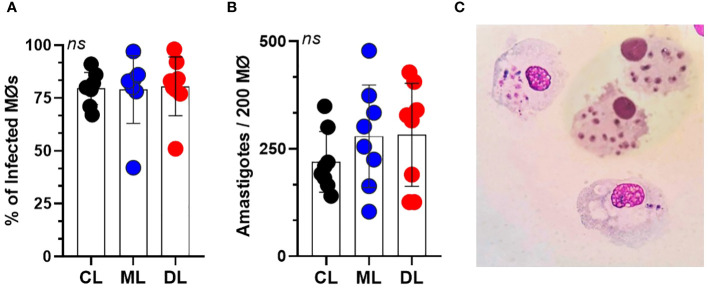
Parasite load of infected macrophages (Mϕ) for 4 hours, at a rate of five parasites per cell. **(A)** Mean percentage of macrophages from 10 healthy donors, infected with each *L. braziliensis* isolate (CL, ML, and DL). **(B)** Average number of amastigotes per Mϕ. There were no differences either in the percentage of infected Mϕ or in the number of amastigotes per 200 Mϕ. Data show mean ± SD. **(C)** Microscopic image of macrophages with internalized *L. braziliensis* parasites (BR/30035).

Macrophages were infected at a ratio of 5:1 with the three isolates used, and the percentage of infected cells after 4 hours was similar for the three isolates of *L. braziliensis* (CL 70 ± 9, ML 75 ± 9, and DL 65 ± 8); **Figure 1A**. The percentage of infected cells and number of amastigotes/200 cells was similar for CL, ML, and DL at all time points (CL 410 ± 121, ML 472 ± 134, and DL 415 ± 151); **Figure 1B**, P>0.05. **Figure 1C** shows a microcopy image of macrophages with internalized parasites derived from patients with CL.

### 3.2 MiRNAs are differentially expressed among ATL isolates

**Figure 2:**
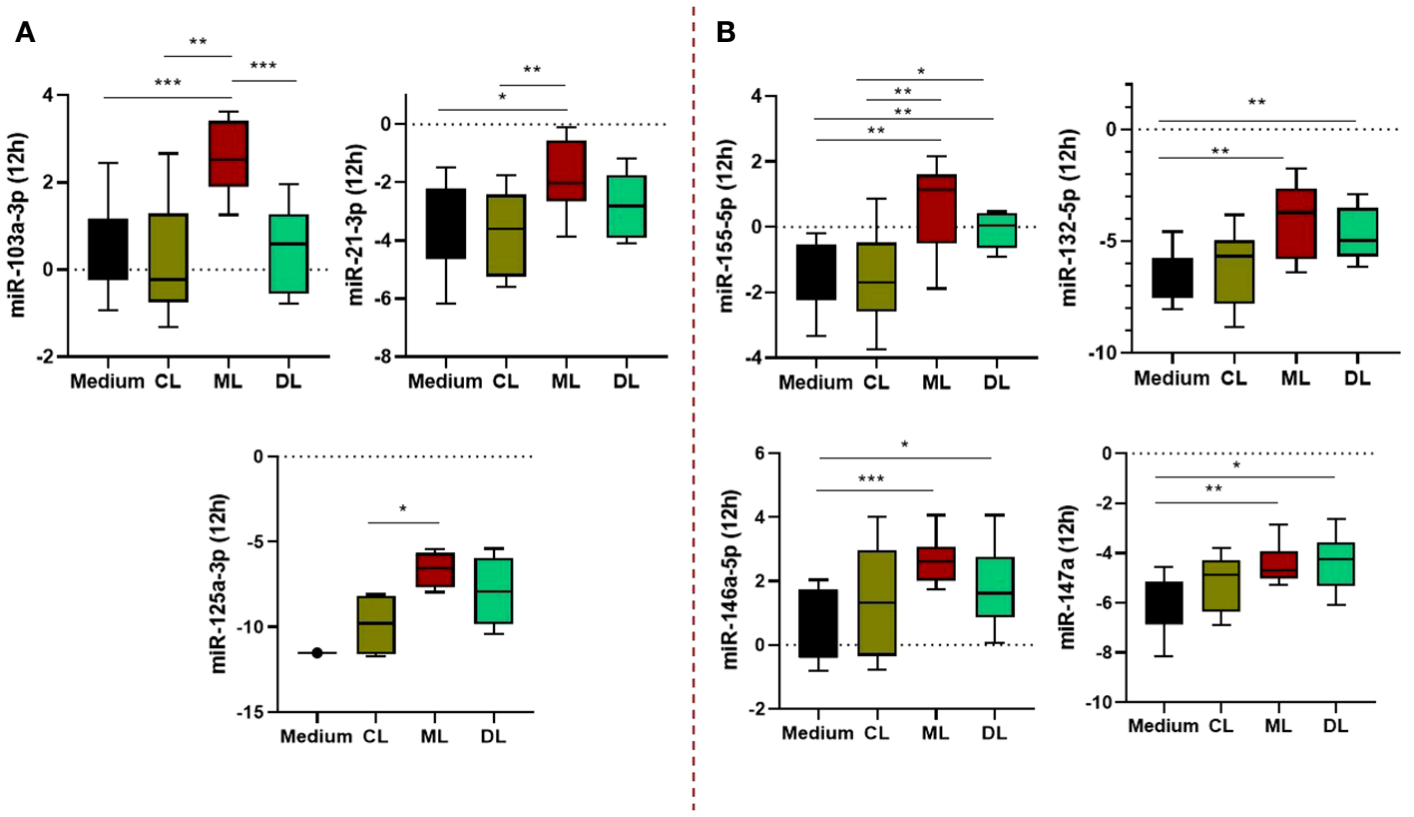
Expression profile of miRNAs regulating TLR/NFkB activation pathways in macrophages (Mϕ) infected with *L. braziliensis* isolates (CL, ML, and DL). The miRs -103a-3p, -21-3p, and 125a-3p were significantly higher in ML than other isolates and uninfected cells **(A)**, while miRs -155-5p, -146a-5p, -132-5p, and -147a, were differentially expressed in Mϕ infected with both ML and DL strains **(B)**. Samples from 10 subjects, in duplicate. After normalization, gene expression was calculated from values of ΔCt. *P<0,05; **P< 0,01; ***P< 0,001.

We evaluated the expression of miRNAs infected with CL, ML, and DL isolates at 4, 12, and 24 hours; see **Supplementary Figure 1**. Only at 12 hours were there differences between isolates. Essentially, these differences occurred between ML and DL in relation to CL and uninfected cells (controls), so all observed associations were for metastatic and invasive forms of ATL. In fact, some of these associations were exclusive to the ML form, such as miRNAs -5103a-3p, -21-3p, and 125a-3p; **Figure 2A**. miRs -155-5p, -146a-5p, -132-5p, and -147a were differentially expressed in macrophages infected with both ML and DL isolates compared to either CL or uninfected, as shown in **Figure 2B**. In addition, we did not observe a correlation between miRNA expression and infection with the CL isolate (data not shown).

### 3.3 MiRNA expressions were correlated with the parasite load in ML

**Figure 3:**
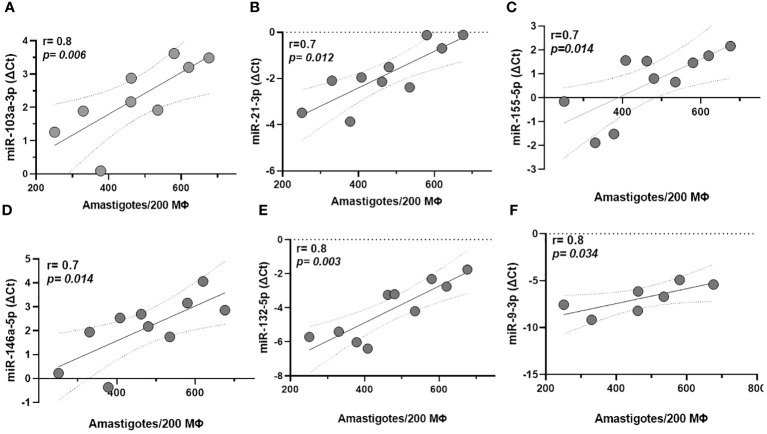
Correlation between miRNA expression and parasite load in macrophages (Mϕ) derived from monocytes infected with mucosal leishmaniasis isolates. MiRNAs -103a-3p, -21-3p, -155-5p, -146a-5p, -132-5p, and -9-3p **(A–F)**, respectively correlate with higher parasitic load in macrophages infected with the ML isolate. Data related 10 subjects with experiments performed in duplicate. Spearman correlation was implemented with GraphPad Prism 8 software. R values above 0.7 and *p*-values < 0.05 were considered significant.

There was a positive correlation between parasitic load in macrophages infected with the ML isolate and miRNAs -103a-3p (r=0.8, p=0.006), -21-3p (r=0.7, p=0.012), -155-5p (r=0.7, p=0.014), -146a-5p (r=0.7, p=0.014), -132-5p (r=0.8, p=0.003), and -9-3p (r=0.8, p=0.034). Interestingly, these represent five of the seven miRNAs that were differentially expressed in this metastatic clinical form, as shown in **Figure 3A-F**. By adding DL, these associations were kept, except for miR132-3p and -9-3p, although the inclusion of this isolate slightly reduced the strength of these correlations, probably due to a greater dispersion of the samples (**Supplementary Figure 2**). We ran the same analysis for CL which returned no significant correlations. On the other hand, the miRs 125a-3 p, -511-3p, let-7a-3p, and 147a returned no significant results for this analysis.

### 3.4 miR-21-3p and miR-146a-5p correlate to antiapoptotic genes and influence cell viability in ML

**Figure 4:**
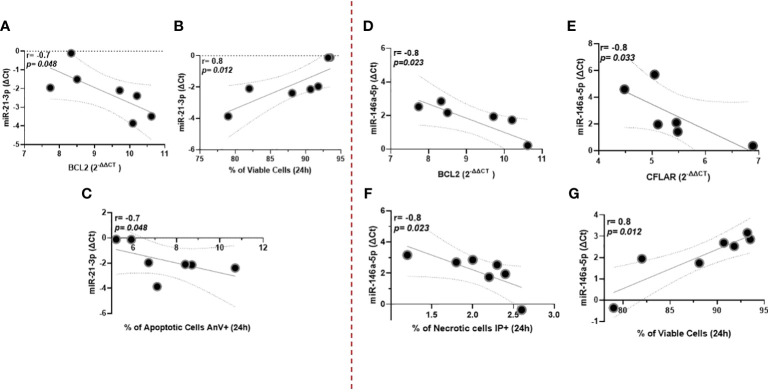
miR-21-3p showed an inverse correlation with the antiapoptotic gene *BLC2*, whereas miR146a-5p was with both *BLC2* and *CFLAR* genes **(A–C)**, respectively. Data related to 10 subjects with experiments performed in duplicate. Both miRNAs were also associated with increased numbers of viable (non-apoptotic) cells in culture for 24 hours **(D, E)**. Additionally, miR-146a-5p correlates negatively with necrotic cells (IP+), whereas for miR-21-3p there is an inverse correlation with apoptotic cells annexin-positive (AnV+) **(F, G)**. Data related to seven subjects with experiments performed in duplicate. Spearman correlation was implemented using GraphPad Prism 8 software. R values above 0.7 and *p*-values < 0.05 were considered significant.

Among miRNAs correlated with parasitic load in ML, we observed that miR-21-3p showed an inverse correlation with the antiapoptotic gene BLC2. In addition, this miR showed an increase in the number of viable cells (non-apoptotic), which was accompanied by an inverse correlation with apoptotic cells annexin-positive (AnV+); **Figure 4A-C**. On the other hand, miR146a-5p was inversely correlated with both BLC2 and CFLAR genes; **Figure 4D, E**. Consistent with this, there was a negative correlation with necrotic cells (IP+) and a positive association with the increase of viable cells; **Figure 4F, G**. **Supplementary Figure 3** shows cell viability and oxidative burst data in the macrophages.

### 3.5 Expression of miRNAs and cytokine production

**Figure 5:**
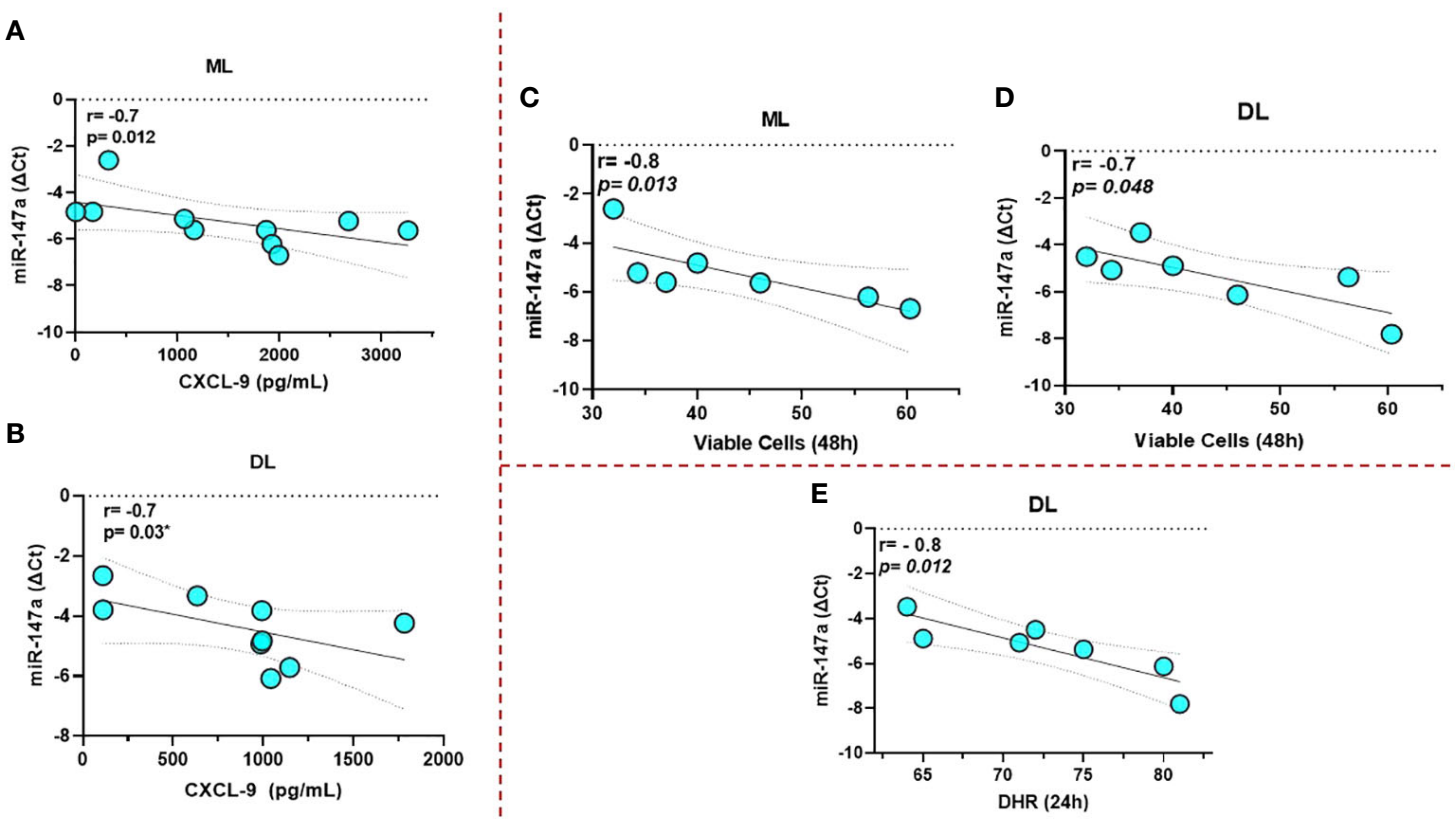
miR-147a showed an inverse correlation with the production of CXCL-9 and the frequency of viable cells for both ML and DL infected macrophages **(A–D)**, respectively. Data related to 10 subjects with experiments performed in duplicate. In addition, for DL, we also observed a correlation with a lower expression of 123 DHR+, indicating an impairment in the oxidative burst **(E)**. Data related to seven subjects with experiments performed in duplicate. Spearman correlation was implemented using GraphPad Prism 8 software. R values above 0.7 and *p*-values < 0.05 were considered significant.

miR-147a showed an inverse correlation with the production of CXCL-9 and the frequency of viable cells in the first hours of infection for both ML and DL infected macrophages; **Figure 5A-D**. In addition, for DL we observed a correlation with a lower expression of DHR+, indicating a parallel impairment in the oxidative burst; **Figure 5E**. None of the other immune markers tested were correlated to the miRNAs tested.

## 4 Discussion

Our study showed expression profile changes in miRNAs from human macrophages infected by different isolates of *L. braziliensis*. Specifically, while isolates from CL did not modify miRNAs expression, isolates from the metastatic forms ML and DL enhanced miRNA expression. These miRNAs share the common feature of regulating TLR activation pathways, participating in events such as lymphocyte activation, cell migration, and cytokine production, in addition to cell death mechanisms. Macrophages, on the other hand, host *Leishmania*, act as antigen-presenting cells, and secrete molecules that induce the inflammatory response and parasite killing (26). MiRNAs -155-5p, -146a-5p, -147a, and -132-5p were significantly more expressed in both ML and DL than CL isolates. Parasites of *Leishmania* spp. are strong promoters of cell-mediated immunity, mainly by induction of pro-inflammatory cytokines after *in vivo* infection. However, during intracellular infection of macrophages, the parasite initially suppresses the signal transduction pathways that lead to these pro-inflammatory responses (27). This likely reflects the parasite’s need to modulate immunity promptly, allowing it to multiply and survive in the host while avoiding excessive responses that could result in its elimination. Our findings strengthen the hypothesis that this modulation is influenced by miRNAs. Consistent with our findings, Lemaire et al. (2013) previously showed that miRNAs -132, -146a, -146b, and -155 were more expressed in macrophages infected by *L. major* (22). In addition, a recent study showed that miR-548d-3p has an important role in CL caused by *L. braziliensis* since inhibition of this miR in infected THP-1 cells was associated with reduced parasite growth early after infection and increased the production of molecules such as MCP1/CCL2, RANTES/CCL5, and IP10/CXCL10, data corroborated in the plasma of individuals with active or cured disease compared to controls (28). Another study showed that during *Leishmania* infection, a signature composed of miR-147a, -146a, -146b, and -155, among others, might influence the inflammatory regulatory network of *L. major*-infected macrophages (29). We propose that once parasites enter the cell, these miRNAs are recruited, assisting the parasites’ ability to multiply and, in some cases, escape from the original site and establish infection in other areas.

Most miRNAs that were associated with ML and DL were also correlated with the parasite load. In leishmaniasis, the activation of macrophages by IFN-γ induces the production of IL-12, TNF, reactive oxygen species (ROS), and nitric oxide (NO) to eliminate the parasite (30). Despite this strong response, enough *Leishmania* survive, and the parasites establish and cause disease. Consistent with this, *L. braziliensis* isolates from DL patients showed a greater ability to penetrate and multiply in monocytes than CL isolates, in spite of a greater respiratory burst induction and increased production of pro-inflammatory cytokines (31). Additionally, monocytes from DL patients were more permissive to parasite infection, with enhanced multiplication compared to cells from CL patients infected with a DL isolate (31). These data show that the combination of the strain, aligned to the host response, determines the parasite load. In lesions from CL patients, *Leishmania* is scarce due to the intense inflammatory response, but the parasite load is quite variable. As ML usually occur after a primary CL, it is expected that in the skin of patients who will develop ML, the parasite load is higher than in those who do not develop ML. We did not observe significant differences in percentages of macrophage infectivity between isolates, but miRNA expression was correlated with parasite load in ML, and this correlation was maintained when we added DL to the analysis. We suggest that the mechanistic details triggered by these parasites, especially in ML, induce a greater expression of micro RNAs that modulate pathways of initial host–pathogen interaction and that, finally, provide parasites with the ability to multiply and escape cells after infection by interfering mechanisms such as phagocytosis, secretion of chemokines and prostaglandins, and the inhibition of IFN-γ inducible pathways.

We observed a negative correlation between the expression of miRNAs 146a-5p and 21-3p and the antiapoptotic *BCL2* gene in macrophages infected with ML isolate, suggesting a correlation between increased expression of these miRNAs, increased *Leishmania* burden, and reduced *BLC2*, which may reflect changes in apoptotic mechanisms by the parasite. This is strengthened by the fact that both miRNAs are also correlated with an increase in viable cells in culture. In addition, miR-21-3p was inversely correlated with apoptotic cells (AnV+) and, similarly, miR-146a-5p with necrotic cells (IP+) after 24 hours. The BCL2 family express domains that are anti-apoptotic, such as BCL-2, MCL-1, and Bcl-xL, and pro-apoptotic proteins such as Bad and Bik. Data showed that functional inhibition of *BCL2* caused increased nitric oxide (NO) response and reduced parasite burden in monocytes after *L. major* infection, confirming the role of *BCL2* during infection (32). Another important apoptosis regulator is *CFLAR*, which inhibits the assembly of caspase-8 and the FADD complex. Increased *CFLAR* expression prevents the accidentally formed signaling complexes between TNFRSF1A/TRADD/RIPK1/FADD, leading to clustering and activation of caspases and therefore apoptosis (33). Our data showed a negative correlation between miR-146a-5p and *CFLAR*, reinforcing a possible role of this miR as an inhibitor of apoptosis. To summarize, we suggest that by inhibiting cell death, the miRNAs help promote the escape and metastasization characteristic of these forms, which would be aligned with their correlation with the parasite load.

The chemokine genes CXCL-9 and CXCL-10 are direct targets of miRNA 147a. In the context of ML and DL, we consider the involvement of this axis in tumor growth and metastasis (34). The function of the CXCL9, -10, -11/CXCR3 axis in response to IFN-γ is mainly divided into two directions: paracrine signaling for immune activation and autocrine signaling for cancer cell proliferation and metastasis. With regard to the paracrine signal, this axis works mainly for the migration, differentiation, and activation of immune cells, including Th1 polarization. On the other hand, for the autocrine signal, cancer cells have a propensity to metastasize due to the activity of tumor-derived ligands mainly through CXCR3A (34). In context, Oliveira et al. (2021) showed that infection with DL isolates causes a different behavior than CL in terms of internalization and multiplication of parasites, as well as activation of monocytes in cells from endemic patients. Yet, according to Oliveira et al.’s study, DL induces more CXCL-9 than CL isolates (31). In addition to CXCL-9, because miR-147a was concurrently associated with lower frequency of viable cells and oxidative burst, we hypothesize that the initial expression of this miRNA may stimulate mechanisms of cell lysis, releasing amastigotes and, finally, favoring a process of dissemination of parasites at some point after infection. We can also assume that inhibition of CXCL-9 by this miR is transitory and restored after time.

As a key part of the regulatory layer of parasite–host interactions, a series of studies have demonstrated the potential of miRNAs as prognostic markers, as well as possible therapeutic targets in cancer and parasitic pathologies such as malaria (35–37), schistosomiasis (38, 39), and Chagas disease (40–42). To date, this is the first study that focuses on different isolates of *L*. *braziliensis* and compares variations in miRNA expression and some of the biological effects, which expands the field and may lead to the identification of new pathways and possible prognostic/therapeutic alternatives based on these molecules in ATL.

## 5 Conclusion

In conclusion, there is a differentiated expression of miRNAs in macrophages infected with isolates of the metastatic ML and DL forms of leishmaniasis. Some of these miRNAs were also correlated with parasite load, genes linked to apoptosis, CXCL-9, and cell viability. We propose that fine regulation by miRNAs in the early hours after infection is the mechanism that favors parasite entry and multiplication in the phagosome to then escape from the original site and establish infection in other areas. Since these miRNAs are involved in events mediated by the activation of TLRs, this regulation involves the modulation of key genes of immune response and cell death.

## Data availability statement

The original contributions presented in the study are included in the article/**Supplementary Material**, further inquiries can be directed to the corresponding author/s.

## Ethics statement

The studies involving humans were approved by Institutional review board of the Federal University of Bahia (CAAE: 93214818.2.0000.0049). The studies were conducted in accordance with the local legislation and institutional requirements. The participants provided their written informed consent to participate in this study.

## Author contributions

LC: Conceptualization, Supervision, Writing – original draft. TL: Investigation, Methodology, Writing – original draft. LM: Methodology. JL: Methodology. NS: Methodology. TC: Formal analysis, Methodology. AR: Methodology. TL-C: Data curation, Formal analysis. EC: Writing – review & editing, Funding acquisition.

## Supplementary material

Supplementary Figure 1 Expression of miRNAs in macrophages infected with different isolates of *L. braziliensis* (CL, ML, DL) at different times of infection (4, 12, and 24 hours). Data is represented in log fold change.

Supplementary Figure 2 Direct correlation between miRNAs -103a-3p, -21-3p, -155-5p, and -146a-5p and infection with the ML and DL isolates in macrophages derived from monocytes.

Supplementary Figure 3 Expression of annexin-positive (AnV+), viable cells (AnV-/IP-), and ROS (123 DHR+) for 24 and 48 hours in macrophages uninfected (medium) and infected with CL, ML, and DL isolates of *L. braziliensis*. Data related to seven subjects, generated by flow cytometry, and analyzed using the FlowJo 7.6.5 program, with minimum of 50,000 gated events from each sample. Kruskal-Wallis test was implemented using GraphPad Prism 8 software.
