# Supplementary figures and images for: MicroRNAs regulating macrophages infected with Leishmania L. (V.) Braziliensis isolated from different clinical forms of American tegumentary leishmaniasis
Source: Front Immunol. 2023 Dec 7;14:1280949. doi: 10.3389/fimmu.2023.1280949 (PMC10748487; doi:10.3389/fimmu.2023.1280949)

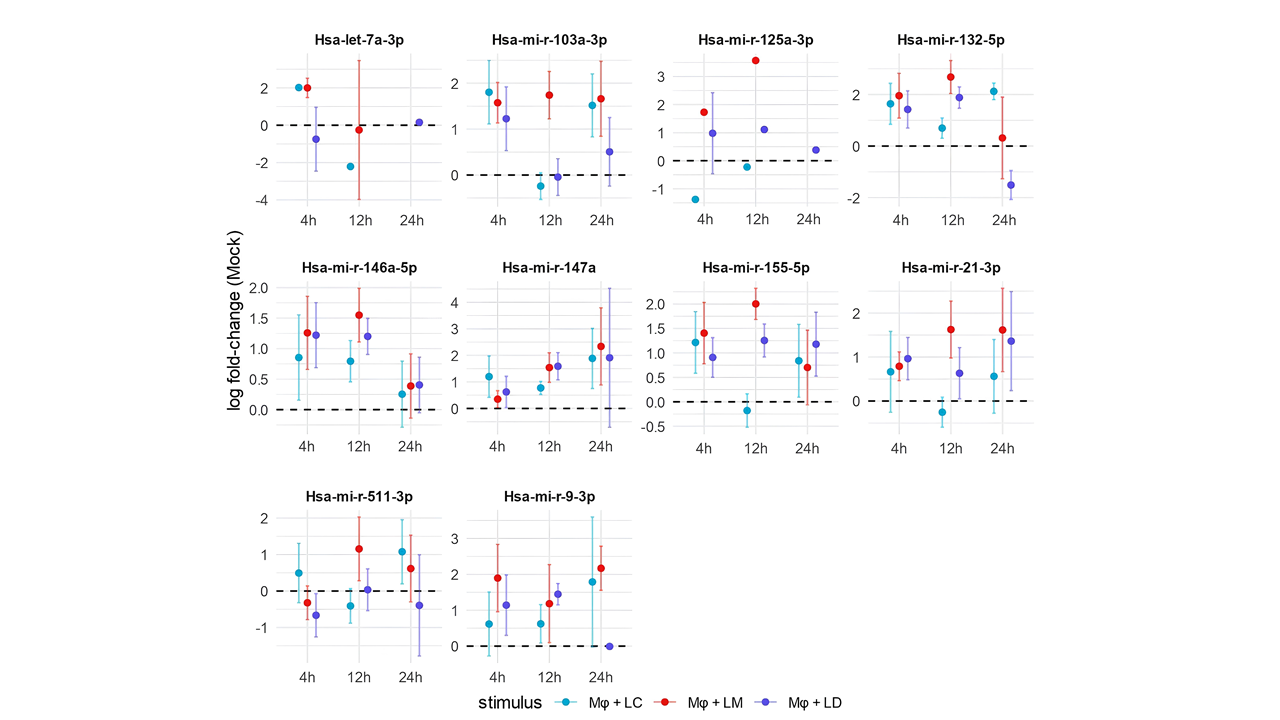

Supplement: Supplementary Figure 1 — Expression of miRNAs in macrophages infected with different isolates of L. braziliensis (CL, ML, DL) at different times of infection (4, 12, and 24 hours). Data is represented in log fold change. [file Image_1.tif]

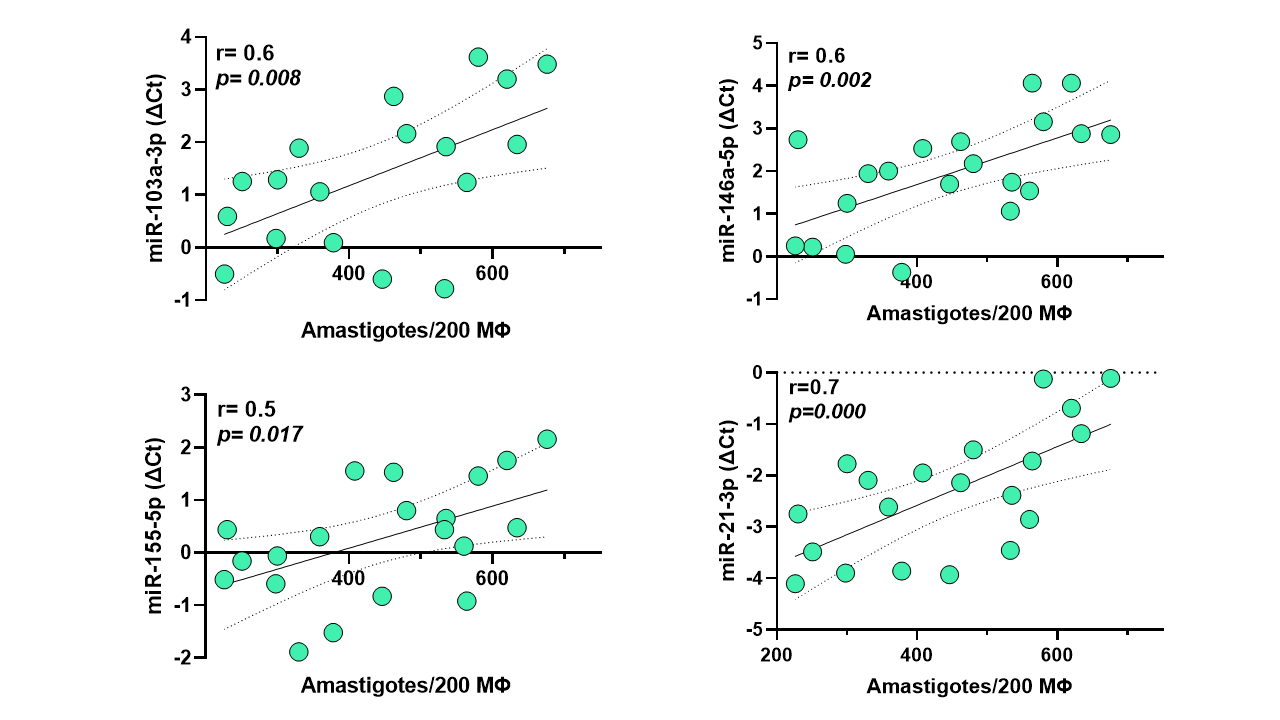

Supplement: Supplementary Figure 2 — Direct correlation between miRNAs -103a-3p, -21-3p, -155-5p, and -146a-5p and infection with the ML and DL isolates in macrophages derived from monocytes. [file Image_2.tif]

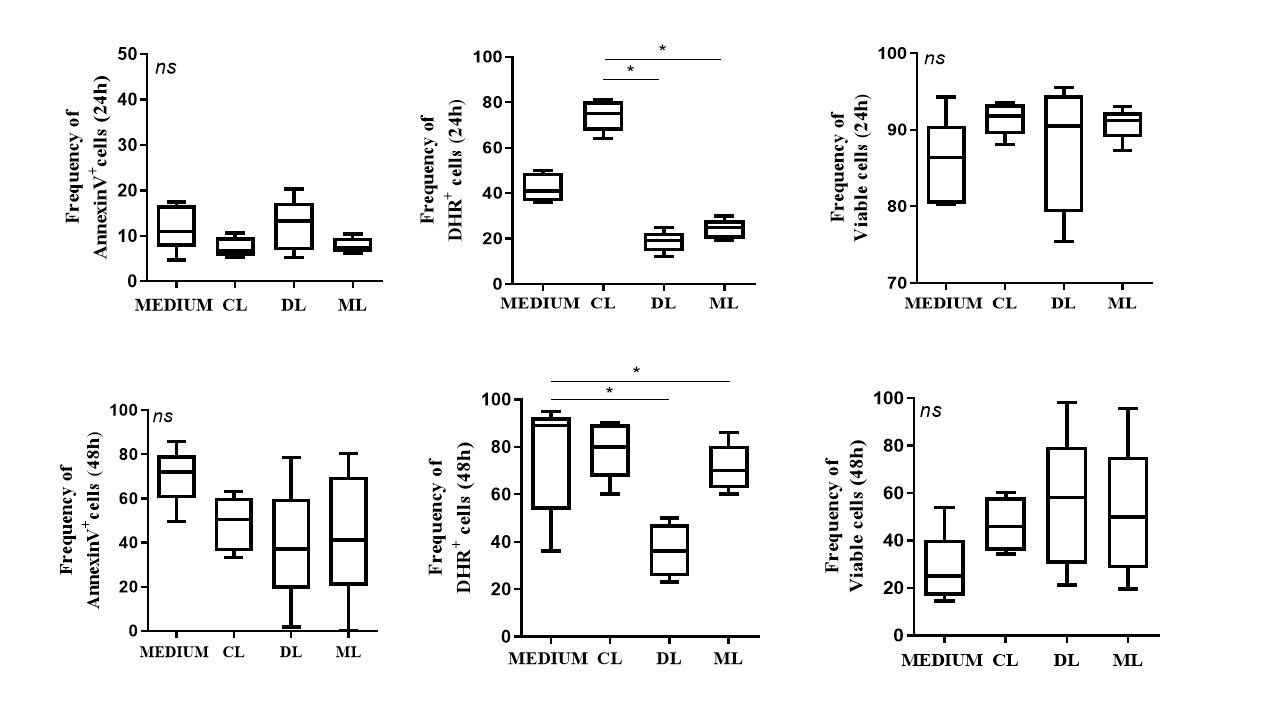

Supplement: Supplementary Figure 3 — Expression of annexin-positive (AnV+), viable cells (AnV-/IP-), and ROS (123 DHR+) for 24 and 48 hours in macrophages uninfected (medium) and infected with CL, ML, and DL isolates of L. braziliensis. Data related to seven subjects, generated by flow cytometry, and analyzed using the FlowJo 7.6.5 program, with minimum of 50,000 gated events from each sample. Kruskal-Wallis test was implemented using GraphPad Prism 8 software. [file Image_3.tif]
